# Temperament Differences

**Published:** 1859-05

**Authors:** 


### TEMPERAMENT DIFFERENCES.

Angel or angel fallen, fills the description of a woman for her life time. For a month before marriage, and a month after death, men (whether many or most, deponent saith not,) regard their wives as angels; all between, angels fallen; the only difference being, that men are angels never. Young ladies before marriage, and widows, strive to make themselves pleasing, and they succeed, the latter especially. After awhile the decree of divorce reads, "on account of incompatibility of temper." But they pleased one another once, and could do it again, and to the end; only the effort is wanting, that tells the whole story. In these times, when a degraded press, uuder the prostituted plea of "liberty," finds a mine of wealth, and consequently gloats over a "divorce case," whether of mechanic or millionaire, whether of the "boards" or the "pulpit," laying before our wives and daughters, morning after morning, the bought-up testimony of outsiders, and the filthy rehearsals of impossible facts by malicious or time serving menials, when these things are so, it becomes those of us who are parents, to consider seriously if something cannot be done to devise safeguards, at least in individual instances, against the calamity of the ought-to-be sacred precincts of the family, invaded by impertinent reporters and lawyers, not less worthy of public execration, who, forgetting that they themselves are husbands and fathers, too often, in the halls of justice, indulge in obscene ribaldries, and disgusting probings; not for truth's sake, but for purposes of sarcasm and of personal abuse, as if right was not to be obtained, without these degrading and infamous aids.

There is a great deal of rank atheism in some of the teachings of phrenology, possibly confined to its blind teachers, in that crime is excused virtually, on account of the "temperament," or "physical conformation." We admit that as far as phrenology teaches and encourages us to rectify these deformities, it is well, but by throwing out intimations, that to a certain extent, crime is excusable on these accounts, making it a moral insanity, it uproots all law, human and Divine, and moral anarchy reigns universal.

We firmly believe, and most unhesitatingly avow, that a sound christianity, early instilled, will uproot this whole matter, and make domestic life akin to angelic communion. The fundamental teaching, the great practical idea of Bible religion, is expressed in two words by its Divine Author, "Deny Thyself." Taking a wider range, it means self control. In its application to all that is desirable, "I can't" is not in the vocabulary of a man, of a christian. That an efficient early self control is possible, is proven in the fact of its practice before marriage; there is nothing in that ceremony which makes it less practicable. The power exists, only the effort is wanting. Let all parents then, let all who are about to be married, let all the married, remember that the inculcation, the practice of self control, is the key stone of practical religion, it is the panacea for domestic disquietude, the entrance door of a heaven on earth, and a brighter heaven in the skies.

On the other hand, let those remember, whether husbands or wives, that the indisposition to make this effort, is not only a want of all religion, it is a want of all nobility, it is beast-like; it demonstrates the want of gentle blood, and the presence of a nature essentially low, one which no improved condition of outward circumstances would ever elevate, but which on the contrary, would be fed by them, and the aids to elevation would be only prostituted to purposes of a deeper degradation. In plainer phrase, the man or woman who will excuse an acknowledged short-coming, by saying "I would do better if I were better off," give me this, that or the other which I have not now, and I will show you that I will be a different being, that person is yet "in the gall of bitterness, and in the bond of iniquity," and needs the pity and the prayer of all good men.

